# Ex Vivo and In Vitro Studies Revealed Underlying Mechanisms of Immature Intestinal Inflammatory Responses Caused by Aflatoxin M1 Together with Ochratoxin A

**DOI:** 10.3390/toxins14030173

**Published:** 2022-02-25

**Authors:** Zi-Wei Wang, Ya-Nan Gao, Sheng-Nan Huang, Jia-Qi Wang, Nan Zheng

**Affiliations:** 1 Key Laboratory of Quality & Safety Control for Milk and Dairy Products of Ministry of Agriculture and Rural Affairs, Institute of Animal Sciences, Chinese Academy of Agricultural Sciences, Beijing 100193, China; wswzw12@163.com (Z.-W.W.); gaoyanan0116@126.com (Y.-N.G.); hsnigot7@163.com (S.-N.H.); jiaqiwang@vip.163.com (J.-Q.W.); 2 Laboratory of Quality and Safety Risk Assessment for Dairy Products of Ministry of Agriculture and Rural Affairs, Institute of Animal Sciences, Chinese Academy of Agricultural Sciences, Beijing 100193, China; 3 Milk and Milk Products Inspection Center of Ministry of Agriculture and Rural Affairs, Institute of Animal Sciences, Chinese Academy of Agricultural Sciences, Beijing 100193, China; 4 State Key Laboratory of Animal Nutrition, Institute of Animal Science, Chinese Academy of Agricultural Sciences, Beijing 100193, China

**Keywords:** aflatoxin M1, ochratoxin A, immature intestine, inflammation, RNA-seq

## Abstract

Aflatoxin M1 (AFM1) and ochratoxin A (OTA), which are occasionally detected in milk and commercial baby foods, could easily enter and reach the gastrointestinal tract, posing impairment to the first line of defense and causing dysfunction of the tissue. The objective of this study was to investigate the immunostimulatory roles of individual and combined AFM1 and OTA on the immature intestine. Thus, we used ELISA assays to evaluate the generation of cytokines from ex vivo CD-1 fetal mouse jejunum induced by AFM1 and OTA and explored the related regulatory pathways and pivot genes using RNA-seq analysis. It was found that OTA exhibited much stronger ability in stimulating pro-inflammatory cytokine IL-6 from jejunum tissues than AFM1 (OTA of 4 μM versus AFM1 of 50 μM), whereas the combination of the two toxins seemed to exert antagonistic actions. In addition, transcriptomics also showed that most gene members in the enriched pathway ‘cytokine–cytokine receptor interaction’ were more highly expressed in OTA than the AFM1 group. By means of PPI network analysis, NFKB1 and RelB were regarded as hub genes in response to OTA but not AFM1. In the human FHs 74 Int cell line, both AFM1 and OTA enhanced the content of reactive oxygen species, and the oxidative response was more apparent in OTA-treated cells in comparison with AFM1. Furthermore, OTA and AFM1 + OTA raised the protein abundance of p50/RelB, and triggered the translocation of the dimer from cytosol to nucleus. Therefore, the experimental data ex vivo and in vitro showed that OTA-induced inflammation was thought to be bound up with the up-regulation and translocation of NF-κB, though AFM1 seemed to have no obvious impact. Since it was the first attempt to uncover the appearances and inner mechanisms regarding inflammation provoked by AFM1 and OTA on immature intestinal models, further efforts are needed to understand the detailed metabolic steps of the toxin in cells and to clarify their causal relationship with the signals proposed from current research.

## 1. Introduction

Mycotoxins are secondary metabolites generated by several range of fungal genera spp., such as *Aspergillus*, *Penicillium*, *Fusarium*, *Alternaria*, and *Claviceps*, which could induce toxic and even carcinogenic outcomes in the human body [1]. Compared to adults, infants are more susceptible to the toxic effects of mycotoxins, for the sake of developing immune, digestive, and detoxification systems, fairly restricted diet, higher rate of metabolism, and lower body weight [2,3,4]. Infants may be exposed to mycotoxins directly by consuming contaminated infant formulae and complementary foods, or indirectly through breast milk as a consequence of maternal exposure [2]. It has been reported that exposure to mycotoxins from breast milk could create a lower risk than direct intake through breast milk surrogates [5]. Besides, the introduction of complementary foods usually occurs earlier than the recommended age of six months in many low- and middle-income countries, which tends to increase mycotoxins exposure during infancy [6,7]. What’s more, early infant co-exposure of mycotoxins from the same or different food sources may also occur, posing additional health risks to the vulnerable population group arising from the synergetic toxicity of some mycotoxins [8,9].

At this critical early stage of development, exposure to mycotoxins, especially aflatoxin M1 (AFM1) and ochratoxin A (OTA), can provoke irreversible damage, ranging from malnutrition, growth retardation, immune dysfunction, and chronic inflammation, to neuro-developmental disorders [10,11,12,13,14,15]. AFM1, the hydroxylated derivative of aflatoxin B1 (AFB1), has been frequently detected in human breast milk and processed infant food samples all over the world [16,17,18,19,20]. According to the latest research, the maximum AFM1 concentrations in breast milk samples from Morocco, Turkey, and southern Ethiopia are 13.33 ng/L, 8.31 ng/L, and 143.3 ng/L, respectively [17,21,22]. Moreover, available data from Mexico showed that 20% of the surveyed commercial infant formulae samples were contaminated with AFM1 in the range of 40–450 ng/L, exceeding the European Commission limit of 25 ng/L [23]. As documented in epidemiological and animal model studies, underweight, stunting, wasting, loss of appetite, hypo-immunity, and intestinal damage have been associated with infancy exposure to AFM1 [24,25,26,27]. Additionally, the high incidence of OTA in cereal-based baby foods and human milk is of increasing concern due to its possible role in causation of future malformation, renal dysfunction, and autism for infants [11,28,29,30,31]. As reported, OTA was found in 47 of 155 infant cereals products from United States ranging from 0.6 to 22.1 ng/g, and all were higher than the maximum limit of OTA in infant food set by the European Commission (0.5 ng/g) [32].

The intestinal epithelium is an integrated single cell layer that selectively permeates nutrients and specific antigens while constituting an efficient barrier against external contaminants [33]. The infant intestine has a relatively high endocytic capacity so as to facilitate the transfer of maternal immunoglobulin and specific antigens from breast milk, thus promoting the normal development of intestinal immune system [34]. However, enhanced intestinal transfer during infancy leads to increased susceptibility to exogenous contaminants such as mycotoxins [34,35]. As reported, ex vivo culture of fetal mouse intestine has been proved to reliably reproduce the in vivo conditions, including inflammatory responses [36,37]. Moreover, human fetal small intestinal epithelial FHs 74 Int cell line derived from normal fetus at 12–16 weeks gestation is more susceptible to inflammatory stimuli than mature enterocytes [38]. Therefore, the aim of this study was to elucidate the effects of AFM1 and OTA treatment alone and in combination on the inflammatory response in the immature intestine through phenotypic toxicity tests and transcriptome analysis and to analyze the interaction effects of the two toxins.

## 2. Results

### 2.1. Effects of AFM1 and OTA Alone and in Combination on the Lactate Dehydrogenase (LDH) and Cytokines Release from the Isolated Jejunal Tissues

**Figure 1:**
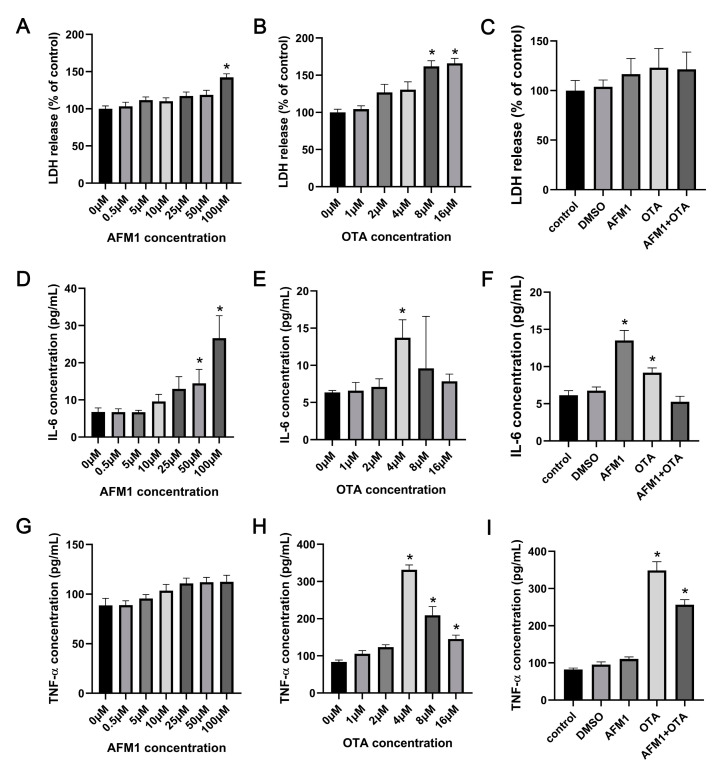
Effects of AFM1, OTA and AFM1 + OTA treatment for 24 h on the release of (**A**–**C**) LDH, (**D**–**F**) IL-6, and (**G**–**I**) TNF-α from the isolated jejunal tissues. In panel (**C**,**F**,**I**), the concentration of AFM1 and OTA was 50 μM and 4 μM, respectively. Results were shown as mean ± SEM (n ≥ 6). * *p* < 0.05 statistically significantly compared with control.

As reflected by the release amount of LDH, AFM1 treatment for 24 h at 100 μM and OTA over 8 μM caused significant cytotoxicity to the tissues (Figure 1A,B). It was seen from the results of ELISA assays that AFM1 higher than 50 μM and OTA of 4 μM significantly induced the release of IL-6 from the jejunum (*p* < 0.05), while AFM1 and OTA at all dosages decreased the level of IL-10 (Figure 1D,E and Supplementary Material Figure S1A,B). Besides, compared to no treatment group, OTA higher than 4 μM significantly increased the concentrations of TNF-α, whereas AFM1 dose-independently increased TNF-α levels without remarkable differences (Figure 1G,H). According to this, OTA exhibited a much higher pro-inflammatory effect than AFM1. As per the principle of inducing the secretion of cytokines but not triggering obvious cell death, AFM1 at the concentration of 50 μM and OTA at 4 μM was selected for ensuing experiments (Figure 1C,F,I). In terms of induction of inflammation, no synergistic interaction between the two toxins was observed. On the contrary, the existence of one toxin seemed to oppose action on the toxicity of the other one (Figure 1F,I), suggesting that antagonism might happen when the two toxins were associated together.

### 2.2. Effects of AFM1 and OTA Alone and in Combination on the Gene Expression Pattern of Jejunal Tissue

**Figure 2:**
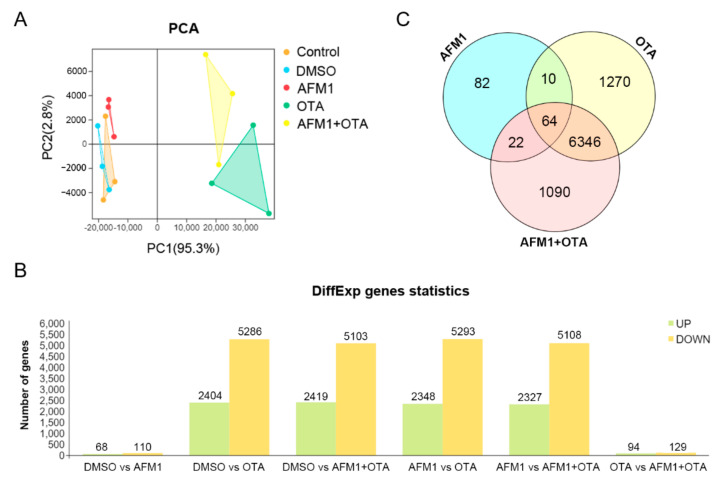
(**A**) Principal component analysis (PCA) plot, (**B**) Number of differentially expressed genes (DEGs) screened out by comparison between groups, and (**C**) Venn diagram plot for transcripts among there toxin treatment compared to DMSO vehicle group.

Via high-throughput RNA-seq analysis, the whole-genome wide expression pattern of each sample was assessed. As shown in Figure 2A, a PCA plot for PC1 and PC2 was established to illustrate the variation between and within each group, in which PC1 captured 95.3% of the total variance and strongly differentiated samples treated with OTA and AFM1 + OTA from those treated with control, DMSO (vehicle blank), and AFM1 treatment. The curve of gene expression abundance (expressed as FPKM) distribution was obviously altered by OTA or AFM1 + OTA (tendency of left-shift indicated relatively low expression of genes globally) (Supplementary Figure S2). Compared to DMSO vehicle treatment, 68 up-regulated and 110 down-regulated genes were found in AFM1 treatment group. For the OTA group, there were 7690 DEGs, including 2404 up-regulated and 5286 down-regulated genes. For the toxin combination group, there were 7522 DEGs, including 2419 up-regulated and 5103 down-regulated genes. Up to 7435 DEGs were discovered between AFM1 and AFM1 + OTA group, in contrast with 223 DEGs between OTA and AFM1 + OTA group (Figure 2B). The OTA group and AFM1 + OTA group overlapped 6410 DEGs, accounting for >70% of total DEGs, but three toxin treatment groups only overlapped 64 DEGs (Figure 2C), which poses a challenge to ordinary enrichment analysis. To sum up, AFM1 slightly changed, yet OTA and the combination of the AFM1 and OTA greatly altered the gene expression pattern and ensuing relevant signaling processes of jejunal cells, might partially contribute to the stimulation of downstream pro-inflammatory factors. Hence, excavating the key pathway(s) and inner sensitive node gene(s) in response to toxins was quite essential in the mechanistic study of toxin-induced inflammation of the fetal intestine.

### 2.3. Pathway Analysis by WGCNA-ORA

**Figure 3:**
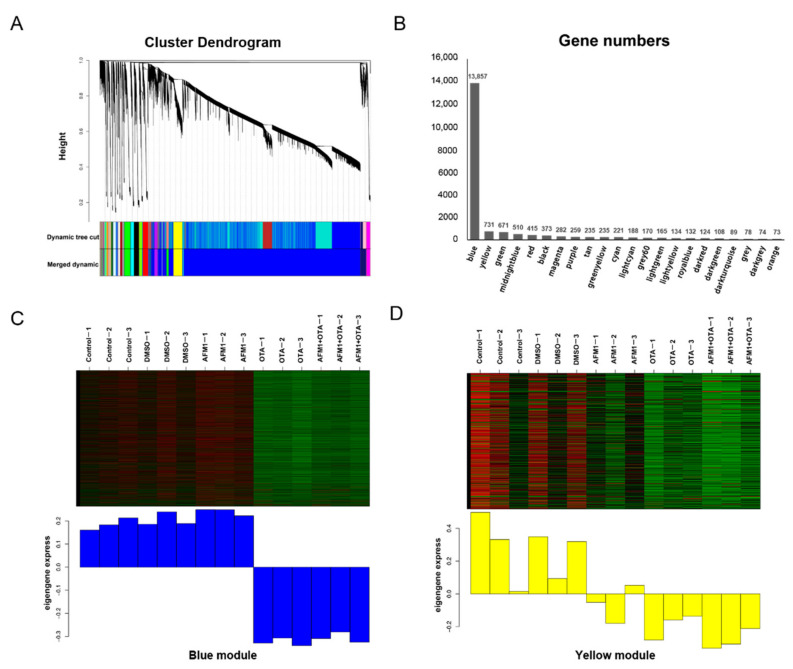
WGCNA analysis to identify groups of genes. (**A**) Clustering diagram showing the co-expression modules recognized by WGCNA. Different colors denote different modules. The longitudinal distance indicates the distance between genes, while the horizontal distance is meaningless. (**B**) The number of genes contained in each module. (**C**) Heatmap of gene expression pattern of blue module. (**D**) Heatmap of gene expression pattern of yellow module.

The strategy of WGCNA, which is not consistent with the principle of manual setting cutoff (fold change and FDR), was applied to choose a cluster of genes with similar expression patterns for subsequent over-representation analysis (ORA). According to the degree of gene co-expression, the whole genes were divided into 22 modules, marked with different colors shown as Figure 3A. A total of 13,857 genes were clustered in the ‘blue’ module and 731 genes were gathered to construct the ‘yellow’ module (Figure 3B). The module eigengene value (weighting of each gene expression in this module) represented the global expression pattern of each sample, and it could be directly seen from Figure 3C,D that ‘blue’ and ‘yellow’ modules had the regular tendency in partitioning different treatments of toxins. Moreover, their Pearson correlation coefficient was 0.69 (*p* = 0.004).

Major genes in ‘blue’ module participated in the courses of ‘translation’ (it was quite regular that cytoplasmic ribosome biosynthesis was activated by OTA, whereas mitochondrial ribosome proteins were almost suppressed by OTA), ‘biological oxidation’ (bioprocess of oxidative phosphorylation and electron transfer chain were somehow enhanced by OTA, but not by AFM1), ‘DNA strand break response’, and ‘cell cycle checkpoint’ (compared with AFM1, more genes conducting DNA repair were detected changing expressions in OTA group) etc. Since the theme of this research was around fetal inflammation, those biological processes in relation to immune responses were preferentially focused on. A total of 52 up-regulated genes were enriched in Reactome ‘cytokine signaling in immune system’ pathway, and 43 in KEGG ‘cytokine-cytokine receptor interaction’ pathway. Likewise, 16 up-regulated genes and 292 down-regulated genes were recognized in the ‘yellow’ module after single and combined toxin treatment. The up-regulated genes were also significantly enriched in the KEGG ‘cytokine-cytokine receptor interaction’ and ‘NF-kappa signaling pathway’, while the down-regulated genes were significantly enriched in the ‘tight junction’ (Table 1). The analytical results above corresponded to the ELISA tests, implying the over-production of cytokines might be controlled by some ‘hub’ genes in particular signal(s) provoked by toxins.

### 2.4. Hub Genes Searching by PPI Analysis

**Figure 4:**
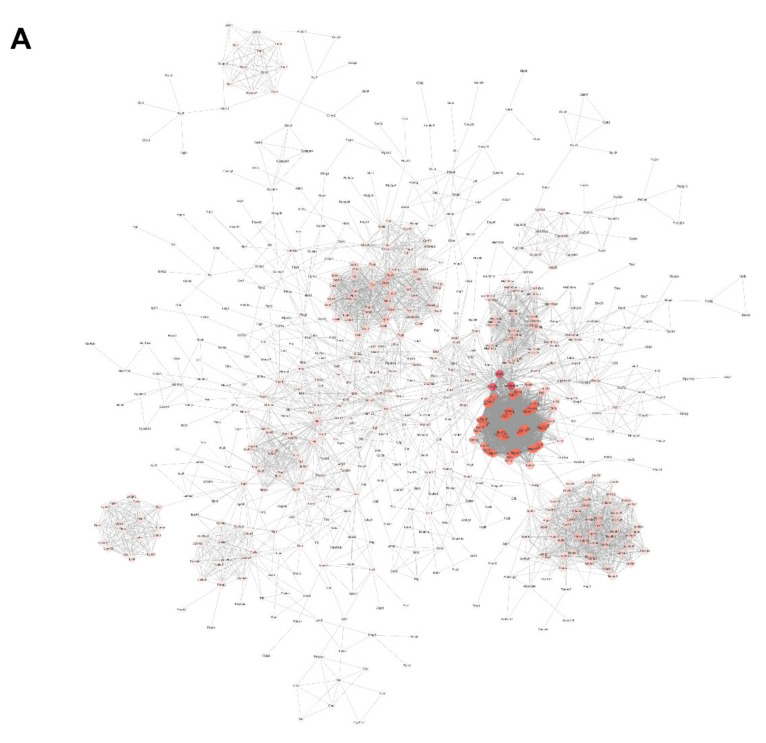

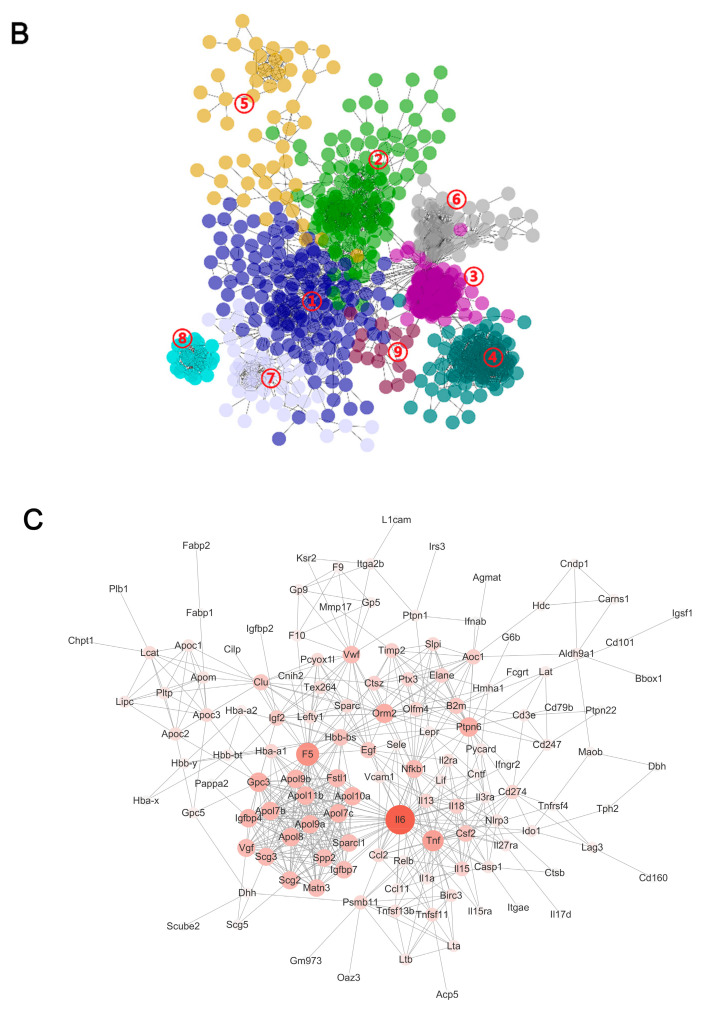
(**A**) Protein–protein interaction (PPI) network analysis of 1788 up-regulated genes in ‘blue’ module plus 16 up-regulated genes in ‘yellow’ module. (**B**) The network was divided into 9 small blocks using ‘Reactome FIviz’ plugin in Cytoscape. Colors represent different blocks. (**C**) PPI network analysis of genes in block 1. In the network, the edges represent gene interactions, and the size and color depth of every node is proportional to the degree of connexity.

A total of 1788 up-regulated genes in ‘blue’ module plus 16 up-regulated genes in ‘yellow’ module in either AFM1, OTA, or AFM1 + OTA group were gathered together and then submitted to the String online database to construct an integrated PPI network. The interaction between gene nodes were regarded to be existed with a confidence > 0.8. Network was visualized by Cytoscape software, and the whole connection was divided into nine small blocks using ‘Reactome FIviz’ plugin in Cytoscape (Figure 4A,B). The gene expression pattern in nine blocks showed good consistency among groups (DMSO, AFM1, OTA and AFM1 + OTA). Of note, the genes in ‘block 1’ were significantly enriched in the ‘cytokine-cytokine receptor interaction’ KEGG pathway and ‘cytokine signaling in immune system’ Reactome pathway (Table 1), suggesting the genes in this block mainly participated in the inflammatory response induced by toxins. The relative expression of each gene involved in this pathway was compared between groups (Supplementary Figure S3), reflecting that most genes (e.g., cytokines production-related genes) were more highly expressed in an effect of OTA in contrast to AFM1.

Then, the genes in block 1 were picked out and again rendered to String PPI network construction. By means of the ‘network analysis’ tool in Cytoscape, the degree of connexity of each gene was calculated, intuitively reflected as the size and color of the node in the diagram (Figure 4C). NFKB1 and RelB, the upstream genes of IL6 and Tnf, attracted the attention referring to previous reports [39,40]. Additionally, the inhibitors of NF-κB, i.e., Nfkbia, Nfkbib, Nfkbie, and Nfkbiz, were all down-regulated to different degrees by OTA and AFM1 + OTA, as shown by RNA-seq. Thus, they were further tested and verified in the following experiments (Section 2.6). However, the transcript level of NFKB1 and RelB was significantly raised by OTA (increased by 2.3-fold and 2.1-fold) and the combination of two toxins (increased by 2.6-fold and 2.5-fold), but not by a single AFM1 treatment.

### 2.5. Effects of AFM1 and OTA Alone and in Combination on the Viability and Cytokines Expression of FHs 74 Int Cells

**Figure 5:**
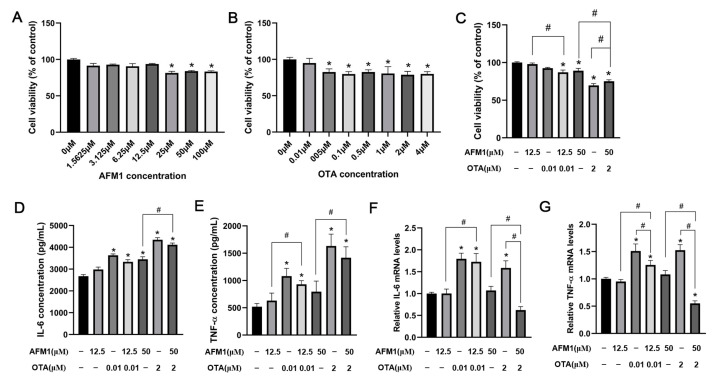
Effects of AFM1 and OTA alone and in combination on the viability and cytokines expression of FHs 74 Int Cells. Cellular viability of FHs 74 Int cells which were treated with (**A**) various doses of AFM1, (**B**) various doses of OTA, (**C**) selected lower and higher doses of AFM1 and OTA alone and in combination for 48 h using CCK-8 analysis. The data of treatment groups are normalized to control (untreated) as the basal 100%. (**D**) IL-6 and (**E**) TNF-α production in the cells treated with toxins were detected by ELISA tests. (**F**) *IL-6*, (**G**) *TNF-α* expression levels in FHs 74 cells detected by RT-qPCR. Gene expression were calculated relative to the internal control (GAPDH) (set as 1.0). Results were shown as mean of three separate experiments ± SEM (n ≥ 6). * *p* < 0.05 significantly compared with control, # *p* < 0.05 significantly compared with combined AFM1 and OTA treatment.

Firstly, CCK assays were performed to assess the viability of FHs 74 Int cells after 48 h incubation with various doses of each toxin, in order to choose the appropriate concentrations in subsequent experiments (Figure 5A,B). Compared with control, OTA of 0.05 μM and higher concentrations remarkably reduced the viability of FHs 74 Int cells (*p* < 0.05), while the lowest cytotoxic concentration of AFM1 was 25 μM. Moreover, for 12.5 μM AFM1 plus 0.01 μM OTA treatment group, a significant (*p* < 0.05) reduction of the cellular viability was observed, even with no significant cytotoxicity recognized in the single toxin treatment group (Figure 5C). Individual AFM1 of 50 μM and OTA of 2 μM caused significant adverse effects on cellular viability (*p* < 0.05), and significant differences were found in their combination as well (*p* < 0.05) (Figure 5C). Thus, for qPCR experiments, 0.01 μM OTA and 12.5 μM AFM1 were selected as the low toxic doses, and 2 μM OTA and 50 μM AFM1 were selected as the high toxic doses.

To evaluate the stimulating effect of AFM1 and OTA alone and in combination on the inflammatory cytokines, ELISA analysis was conducted to measure the IL-6 and TNF-α concentrations (Figure 5D,E). Compared to control, the release of IL-6 but not TNF-α was significantly increased in the cells treated with high toxic doses of AFM1 (*p* < 0.05). Moreover, OTA and AFM1 + OTA at low and high toxic doses all caused a remarkable increments of IL-6 and TNF-α in contrast to control (*p* < 0.05). Afterwards, the gene expression of *IL-6* and *TNF-α* in FHs 74 Int cells after toxins treatment were tested. OTA at low and high concentrations both stimulated the mRNA level of *IL-6* and *TNF-α* (Figure 5F,G). It was noteworthy that when blended, AFM1 + OTA seemed to have an anergic action instead of positive-going association on the gene expressions of *IL-6* and *TNF-α* (Figure 5F,G), and the combination of AFM1 and OTA also had a trend to reduce the cytokine-releasing induced by single OTA (although not statistically significant) (Figure 5D,E). Besides, *IL-6*, *TNF-α*, *NFKB1*, and *RelB* were selected to conduct qPCR assays in FHs 74 Int cells to validate RNA-seq results. Intriguingly, notwithstanding different species of model used in this study (human intestinal cell line and mouse intestinal tissue), the results of RT-qPCR of FHs 74 Int showed good reproducibility compared with the transcriptome data of intestinal tissue from fetal mice (Supplementary Figure S4).

### 2.6. NF-κB Acted as a Key Sensor in the Process of Inflammation Induced by OTA and AFM1 + OTA

**Figure 6:**
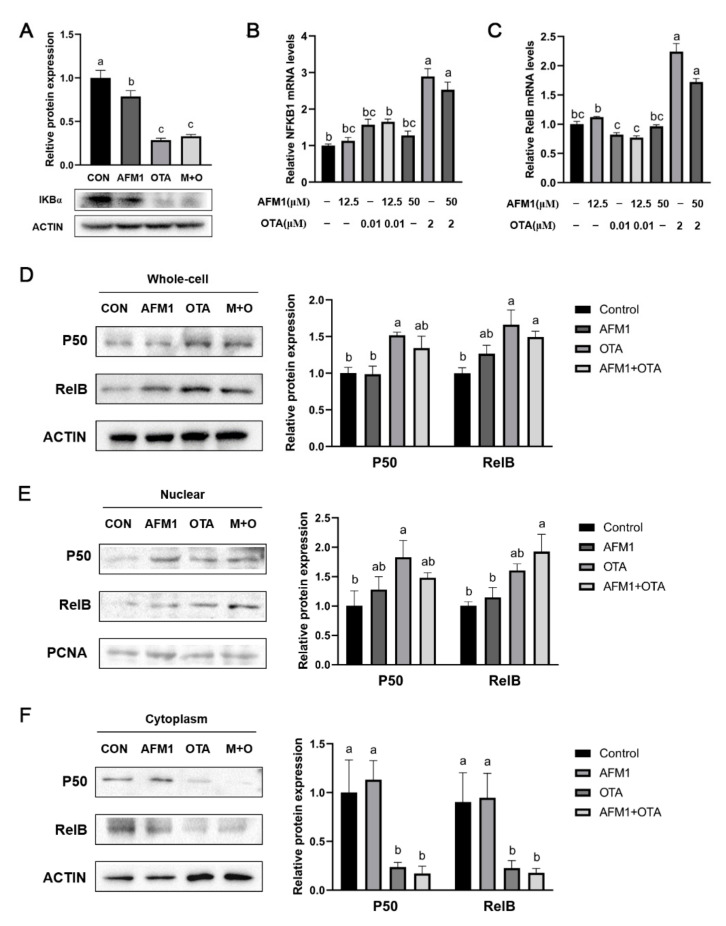
Effect of single and combined AFM1 and OTA on IκBα degradation and NF-κB activation in FHs 74 Int cells. (**A**) Relative protein expression levels of IκBα (n = 3). (**B**) *NFKB1*, and (**C**) *RelB* mRNA expression levels in FHs 74 Int cells detected by RT-qPCR (n = 6). Relative protein expression of p50 and RelB in (**D**) total protein, (**E**) nuclear protein, and (**F**) cytoplasmic protein of cells (n = 3). The bands of IκBα, p50 and RelB were detected by western blotting and quantitated by density analysis tool of Image J software. The data was represented as mean ± SEM. All values in treatment group were compared and normalized to control (set as 1.0). Different lowercase indicates statistical differences between groups (*p* < 0.05).

On account of NFKB1/RelB being closely linked to the activation of Tnf and IL6, the degradation of IκBα as well as the abundance and cellular location of NFKB1/RelB heterodimeric complex in FHs 74 Int cells was tested. As indicated in Figure 6A, OTA and AFM1 combined with OTA significantly induced IκBα degradation (*p* < 0.05), which could provoke the nuclear translocation of NF-κB. Results of RT-qPCR analysis showed that high dosage of OTA stimulated gene expression of *NFKB1* and *RelB*, while AFM1 did not have any obvious impact (Figure 6B,C). According to Western blotting results, total expressions of the two proteins were significantly up-regulated by OTA (2 μM) and AFM1 + OTA (50 μM + 2 μM) treatment (*p* < 0.05), while AFM1 (50 μM) had no effect (Figure 6D). Moreover, the contents of NFKB1 and RelB were both obviously reduced in the cytosol but lifted in the nucleus by OTA and AFM1 + OTA treatment (*p* < 0.05, Figure 6E,F), indicating that the p50/RelB dimer was translocated to nucleus, where it might act as the activator of transcription to downstream gene(s) (e.g., IL-6 and TNF-α), promoting their expression levels.

### 2.7. ROS Mediated Inflammatory Responses Induced by OTA and AFM1 + OTA

**Figure 7:**
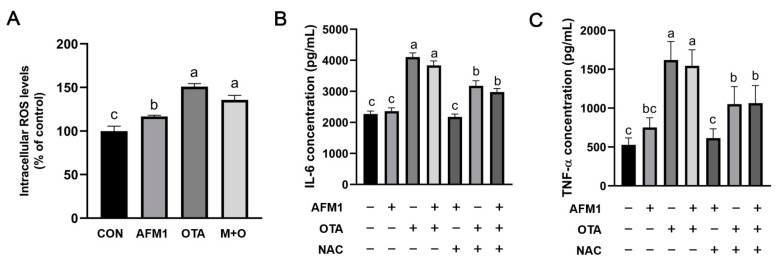
(**A**) Effects of individual and combined AFM1 and OTA on ROS production in FHs 74 Int cells for 48 h (n = 3). The data of treatment are normalized to control (untreated) as the basal 100%. (**B**) Effects of ROS inhibitor NAC on the IL-6 production in cells treated with toxins for 48 h (n = 6). (**C**) Effects of ROS inhibitor NAC on the TNF-α production in cells treated with toxins for 48 h (n = 6). The data was represented as mean ± SEM. Different lowercase indicates statistical differences between groups (*p* < 0.05). Con represents the untreated group, AFM1 represents AFM1 at 50 μM, OTA represents OTA at 2 μM, and M + O represents 50 μM AFM1 + 2 μM OTA, NAC represents NAC at 5 μM.

To investigate the possible role of ROS in the inflammatory responses evoked by single and combined AFM1 and OTA, intracellular ROS levels in FHs 74 Int cells were determined firstly. As shown in Figure 7A, OTA and AFM1 + OTA dramatically increased the intracellular ROS production (*p* < 0.05), and the oxidative response was more apparent in OTA-treated cells in comparison with AFM1. Moreover, *N*-acetyl-cysteine (NAC), a ROS inhibitor, significantly reversed the increased IL-6 and TNF-α levels in cells treated by OTA and AFM1 + OTA (*p* < 0.05, Figure 5B,C), implying that intensive oxidative stress might be one of the mechanisms by which OTA exhibited strong pro-inflammatory action on immature intestinal cells.

## 3. Discussion

The intestine contains 70–80% of human immune cells and tissues and is regarded as the first line of defense against potentially harmful substances. Infancy is the critical period for the maturation of intestinal immune barrier that plays a fundamental role in the development and function of the immune system [41]. Diet-derived contaminants including mycotoxins may change the gut microbiota composition and induce intestinal inflammation during infancy [42], associated with higher risk of allergies, inflammatory bowel disease, and autoimmune diseases later in life [43,44,45]. A large number of researches have indicated that mycotoxin exposure can provoke inflammatory response in mature intestine models, which is manifested as imbalanced inflammatory cytokines, enhanced oxidative stress, and obvious inflammatory cell infiltration [46,47,48,49,50]. However, there is scant literature on the inflammatory injury caused by mycotoxins in immature intestinal models. In the present study, human fetal small intestinal epithelial FHs 74 Int cell line and ex vivo culture of fetal mouse intestine were used for the first time to detect the inflammatory response to individual and combined AFM1 and OTA treatment.

OTA was reported to result in the secretion of pro-inflammatory cytokines such as IL-6, TNF-α, IL-1β, etc., and affect immune function in the intestine, which corroborated the current study [51,52]. The immunity-related researches of AFM1 were scarce, though another aflatoxin (AFB1) was extensively studied. In breast milk and infant food, AFM1 had higher frequency to be detected than AFB1 [2], and accordingly, to be considered here. It was shown that OTA exhibited much stronger ability in inducing the releases of pro-inflammatory factors than AFM1 using immature intestinal models. Whether or not they both executed the same cellular biological processes needed deep discovery on the molecular scale.

In view of the production of various cytokines and chemokines being modulated by the expression of their own genes, the classical methodology is applying transcriptomics analysis on the whole-genome wide range to search the genes differentially altered by AFM1 and OTA [53,54]. In this study, RNA-seq data from ex vivo fetal jejunum conformed to the phenotype of ELISA tests, while the objective of filtering a specific gene population closely related to fetal intestinal inflammation always relies on the analysis of the biological pathway. The central prerequisite is that a series of genes in a certain route (from upstream to downstream) conducting the same functional effects are mostly affected by toxins, rather than a single or a few genes being involved [55]. The subsequent task for searching one or several hub gene(s) was based upon the result of pathway analysis and inner connection of the PPI network. This flow of strategy that narrowed the scope of candidate genes step-by-step is just like stripping off the orange peel. For example, Zhang et al. (2020) identified hundreds of DEGs in porcine intestinal epithelial cells (IPEC-J2) triggered by deoxynivalenol (DON). The MAPK-related immune pathway was significantly enriched and linked to the motivation of cellular inflammation. Then, p38 and ERK1/2 were further proven to be the pivot genes in the pathway, and the inhibition on the two genes attenuated the over-expression of IL6, TNF-α, CXCL2, CXCL8, etc. [56]. Likewise, Yang et al. (2020) acquired >10,000 DEGs from transcriptome analysis in OTA-induced differentiated Caco-2 cells. Followed by ORA and insight of PPI network, downregulation of gene expression of MDM2 and upregulation of Noxa and CASP3 were considered to be partly attributed to cellular apoptosis, causing impairment to the intestinal function [57].

In this study, WGCNA, a systems biology method for calculating the correlation similarity of expression among genes across multiple samples and allocating genes into different clusters (modules), was used to screen out the core biological processes aroused by AFM1 and OTA. Then, the vital node genes involved served as the responder or transducer were speculated and tested. Finally, NFKB1 and RelB were recognized as hub genes through PPI network analysis (Figure 4C), and cell experiments verified the up-regulation and translocation of NF-κB (p50 and RelB) in OTA and AFM1 + OTA treatment group (Figure 6). The p50 (NFKB1)/RelB dimer, despite not being a common dimeric association (such as p50/RelA in canonical pathway, and p52/RelB in non-canonical pathway), was also shown to transcriptionally activate homeostatic chemokines CCL19 in dendritic cells (DCs) stimulated with TNF-α or LPS [58,59]. Similarly, Moriwaki et al. (2014) found that receptor interacting protein kinase 3 (RIPK3) specifically promoted the transcriptional level of several cytokines (IL-23, IL-1β, TNF, and MCP-1) in LPS-treated DCs through RelB-p50 activation and nuclear translocation [60]. Many previous references already addressed that mycotoxins, including OTA, AFB1, DON, etc., stimulated NF-κB pathway in their actions of exerting pro-inflammation, oxidative stress and intestinal barrier injury [46,52,61,62]. However, the evidences from current transcriptional analysis were not adequate to prop up the causal relation between OTA and NF-κB leading to inflammation in fetal intestine. It was plausible that carrier systems like the H^+^-dipeptide cotransporter, the organic anion transporter (OAT), and the organic anion transporting polypeptide (OATP) were implicated in OTA across cells, notwithstanding some contradictions announced as well [63]. Since the path of OTA be absorbed into intestinal cells is elusive till now, it was herein not convincing to conclude the over-releases of cytokines followed by the treatment of toxin was directly passing-through NF-κB. Besides, whether the difference of AFM1 and OTA in facilitating cytokines is mainly attributable to the extent of stimulation of NF-κB was far from clear based on our results for the moment. Hence, more explorations are needed to enucleate the toxicokinetics of AFM1 and OTA in fetal intestinal cells. Here, we proposed some underlying mechanistic hypotheses referring to the results obtained from the present research and previous reports.

(I) Toxin-derived reactive forms of by-products and intermediates affected NF-κB-related inflammation. Except for the liver and kidney, the major organs of the metabolization and accumulation of mycotoxins, the intestinal mucosa also has the capacity to bio-transform AFM1 and OTA on account of a diversity of enzymes: CYPs (P450), prostaglandin synthase (PGSH), and lipoxygenase (LOX), etc. [64]. AFM1 can be metabolized to AFM1-8,9-epoxide and aflatoxicol M1. Under complex metabolic systems, OTA could be converted to different forms: OTα, ochratoxin B (OTB), ochratoxin C (OTC), epimers of 4-hydroxyochratoxin A (4-OH OTA), 10-hydroxyochratoxin A (10-OH OTA), lactone-opened OTA (OP-OTA), and OTA-derived quinones (OTAQ/OTAHQ). Such biotransformations were regarded as detoxification or bioactivation steps to AFM1 and OTA [65]. Besides, conjugation reactions of OTA and AFM1 with endogenous glucuronic acid, sulfate, and glutathione (GSH) occurred synchronously [63,66]. All of the processes changed the running sides of mycotoxicoses of AFM1 and OTA. In particular, OTA and some of its metabolites exhibited intensive activity to elicit oxidative stress by over-producing reactive oxygen species (ROS) detected by electronic paramagnetic resonance spectroscopy [67], impairing the antioxidant defense systems (such as depletion of GSH, inactivation of antioxidative enzymes, and inhibition of Nrf2-related genes) [68,69], and thus exerted pro-inflammatory effects indirectly [65]. To date, numerous researches have revealed the toxicology of aflatoxins and ochratoxins were somehow attributable to the toxin-induced disturbances of redox homeo-stasis [65,69,70]. Forrester et al. (2018) and Shi et al. (2003) confirmed that accumulative ROS, when not to be scavenged in time, could irritate NF-κB and further arouse the productions of cytokines [71,72]. As demonstrated in our results, AFM1 and OTA both disturbed the oxido-reductive balance in FHs 74 Int intestinal cells, which might be one of the mechanisms implicated in the degree of occurrence of inflammation. In addition, toxins together with excessive ROS could cause damage to DNA (double strand-breaking and DNA-adducts forming), lipid peroxidation, alteration of permeability of membrane, and disruption of calcium homeostasis and be involved in promoting the programmed cell death (PCD) and necrosis [73,74]. The LDH assay in our research laterally showed that OTA did great physical damage to immature intestine tissue compared with AFM1. Some damage-associated molecular patterns (DAMPs), which are the substances secreted or exposed by injured or necrotic cells, could be recognized by pattern recognition receptors (PRRs) of cells, thereafter inspiring the NF-κB pathway and inducing inflammatory mediators [39].

To partially support this, we selected and listed several proximal genes to NFKB1 from the network (Supplementary Table S2), so as to conjecture and comprehend the role of NF-κB in the way of toxin-induced inflammation. Among these genes, NLRP3 attracted our concerns due to its gene expression being up-regulated in AFM1, OTA, and AFM1 + OTA groups, and the extent of the increment was higher in OTA and AFM1 + OTA than AFM1. NLRP3 belongs to NOD-like receptor (NLRs) family which is one of the most important members in PRRs regarding innate immune response [75]. The protein of NLRP3 mainly locates in nucleus and cytosol, and leads the assembly of inflammasome, together with adapter protein ASC (gene name: *PYCARD*) and pro-caspase 1 [76]. The expression of *PYCARD* and *Casp1* were lifted by OTA and AFM1 + OTA. Upon stimulation by diverse events, such as mitochondrial dysfunction, oxidative stress, and deregulation of calcium flux, NLRP3 recruits pro-caspase 1 via ASC, thereby processing pro-caspase 1 to the bioactive protein [76]. Activated caspase 1 then cleaves pro-IL1 and pro-IL18 into their mature forms, which further trigger cascades of cellular inflammation [77]. Intriguingly, in our study, the gene expression of IL-1β was increased by AFM1, but not by OTA. Meanwhile, the expression of IL-1α and IL-18 was increased by OTA, but not by AFM1, indicating that some unknown manipulations above were separately controlled by AFM1 and OTA during their mycotoxicosis. Of note, dysregulation of NLRP3 inflammasome was mediated by active NF-κB (NLRP3 is a direct target gene of NF-κB and contains NF-κB-binding sites in its promoter region), and there have been compelling evidences showing that NLRP3 inflammasome via activation of NF-κB contributed to the onset of inflammatory bowel disease (IBD) [78,79]. As aforementioned, excessive ROS and other ensuing substances (e.g., DAMPs) brought by toxins (especially for OTA) could call up the signal transduction to NF-κB. It was further found that the replenishment of antioxidants declined the gene expression of *NFKB1* and allayed the degree of inflammation in toxin-treated intestinal cells, providing evidence that ROS might be served as the second messenger of OTA in regulating inflammatory responses, whereas AFM1 induced weaker pro-inflammatory responses as a result of limited ability in ROS generation.

(II) Specific interaction between toxin with a target molecule (e.g., receptor), triggering downstream NF-κB by programming signal transduction cascades. We put forward speculation that ROS scavengers could not entirely restore the level of cytokines. Hence, the above explanations might be one-sided to uncover the mechanisms of phlogistic processes caused by toxins. Zearalenone (ZEN), another toxin from *Fusarium graminearum*, was verified to pose reproductive adverse effects and inflammatory consequences after binding to estrogen receptors, ERα and ERβ, and be responsible for the stimulation of gene of NF-κB [80]. Liu et al. (2019) proved aryl hydrocarbon receptor (AhR) played a key role in the transcriptional activation of CYP1A5 by T-2 toxin, thereafter reducing cell viability, causing oxidative stress and inducing DNA damage as well as apoptosis [81]. The methodology of transcriptome profiling could not let us get detailed information discerning the interaction between OTA and certain molecule(s) (if any). Whether OTA and its cellular metabolites have corresponding receptor(s) (on the membrane surface, or in the cytoplasm or nucleus of fetal intestinal cells) with high affinity, which possibly conduces to the activation of NF-κB, deserves next investigations. In view of the analysis of ‘structure–activity relationships’ (SAR), OTA and most of its metabolites (except for OTα) commonly contain the moiety similar to phenylalanine (Phe) that can act as a substitute for Phe, which might facilitate conjunction with macromolecules or impede the synthesis of proteins, finally impacting on the expression of NF-κB [65]. Given that NF-κB played a pivotal role in the toxin-enhanced inflammation of the intestine, AFM1 seemed to have a negligible influence on its expression and location. To figure out a clear intracellular metabolic route of AFM1, it is a convenient approach to adopt in silico analysis by means of molecular docking to predict and filter the possible targets of AFM1. In addition, it might be feasible to use the antibody against OTA or AFM1 to capture their linked proteins in cells (similar to the method of co-IP), and to use in vitro tests such as SPR or ITC to measure the binding constant of toxins with specific macromolecules.

Another vital issue with regard to the spatial interactions of AFM1 and OTA in current study, namely, why mixture of AFM1 and OTA represented lower pro-inflammatory effects compared to individual OTA treatment, could be answered on the basis of the above conceptions. The synergism of different toxins always relies on exerting toxicity by means of the same path or one toxin promoting the rate of absorption of the other. On the contrary, antagonistic action happens when competing for the same target. Hence, AFM1 and OTA might share a common channel of transporting into intestinal cells, or probably, their way of executing pro-inflammation is similar but conflicting [82]. Tavares et al. (2013) utilized mathematic model to estimate the cytotoxic relationship of AFM1 and OTA and found that both of the toxins at low concentration presented an antagonistic association owing to a competition for glutathione and thus decreased the level of ROS produced by single OTA-brought redox cycling [83]. More detailed investigations are required in this respect in the future.

To sum up, we exploited phenotypic assays to depict and compare the inflammatory responses driven by AFM1 and OTA, which is the first attempt launched in the fetal intestine. Transcriptomics analyses gave an overall impression concerning the genomic expression scale to uncover the latent intracellular molecular changes and to decipher related mechanisms. A better understanding of key pathways and genes that are related to mycotoxin-induced inflammatory injury of the fetal intestine is of great significance for the development of detoxification schemes with appropriate and accurate targets. However, many other unsolved questions remain because protein translation and post-translational modifications can be altered by AFM1 and OTA. Moreover, elaborated toxicokinetics reflecting metabolic courses of toxins in cells and inner relationships with intracellular signals are poorly known. In the long run, further substantial valuable efforts are still needed.

## 4. Materials and Methods

### 4.1. Chemicals and Reagents

Standard AFM1 and OTA (purity greater than 99.0%) used in the experiment were purchased from Pribolab (Singapore) and then dissolved in Dimethylsulfoxide (DMSO) to prepare 10 mg/mL stock solution stored at −20 °C. For isolated tissue ex vivo culturing, Opti-MEM I medium, HEPES buffer, L-glutamine, and fetal bovine serum (FBS) were purchased from Gibco (Grand Island, NY, USA). Hydrocortisone was purchased from MedChemExpress (Monmouth Junction, NJ, USA). Recombinant mouse epidermal growth factor (EGF) and ITS liquid media supplement (100×) were purchased from Sigma-Aldrich (St. Louis, MO, USA). For cellular in vitro culturing, human fetal small intestinal epithelial FHs 74 Int cell line and its corresponding basal medium (Hybri-Care Medium) were purchased from American Type Culture Collection (ATCC) (Manassas, VA, USA), and recombinant human EGF was from Sigma-Aldrich (St. Louis, MO, USA). For determination assays, the LDH cytotoxicity assay kit, the enhanced cell counting kit-8 (CCK-8), and BCA protein measuring kit were purchased from Beyotime Biotechnology (Shanghai, China). Enzyme-linked immunosorbent assay (ELISA) kits quantitating the contents of mouse Interleukin (IL)-6, TNF-α, and IL-10 were from R&D Systems, Inc. (Minneapolis, MN, USA). Human IL-6 and human TNF-α ELISA kits were from Abcam (Cambridge, UK). Reactive oxygen species (ROS) assay kits were obtained from Solarbio (Beijing, China). Reagents for Western blotting, including lysis buffer, protease inhibitor cocktail, TBS with 0.1% (*v*/*v*) Tween-20 (TBST), and Western blocking buffer, were purchased from Solarbio (Beijing, China). The primary rabbit-sourced antibodies against β-actin, PCNA, NFΚB1, RelB, and secondary antibody ‘goat anti-rabbit IgG/HRP’ were purchased from Bioss (Beijing, China), and Pierce™ ECL Western Blotting Substrate was purchased from Thermo Scientific Fisher (Waltham, MA, USA).

### 4.2. Ex Vivo Culturing of Isolated Jejunal Tissues

All animal experiments were approved by the Ethics Committee of Institute of Animal Sciences, Chinese Academy of Agriculture Sciences (Beijing, China) under protocol number IAS2019-3. CD-1 mice (from Beijing Vital River Laboratory Animal Technology Co., Ltd., Beijing, China) were raised in the Specific Pathogen Free (SPF) barrier system on a 12/12 h light and dark cycle, with the ambient temperature at 25 ± 2 °C, and relative humidity at 55 ± 5%. They were acclimated to the environment for 7 days, and then mated. The day a copulation plug was observed was considered the 1st day of pregnancy. After that, fetal mice were removed by C-section on day 18 of gestation, and 3 mm long pieces of jejunum tissue were then collected, washed, and maintained in Opti-MEM I medium supplemented with 10% (*v*/*v*) FBS, 1% (*v*/*v*) ITS Liquid Media Supplement, 2 mM L-glutamine, 200 nM Hydrocortisone, 10 mM HEPES buffer, 200 ng/mL recombinant mouse EGF, and antibiotics (100 U/mL penicillin, 0.1 mg/mL streptomycin, and 0.25 μg/mL Amphotericin B) as previously reported [84].

### 4.3. Treatment of Isolated Jejunum Tissues with Individual and Combined AFM1 and OTA

The jejunal tissues were treated with AFM1 (0, 0.5, 5, 10, 25, 50, 100 μM) or OTA (0, 1, 2, 4, 8, 16 μM) for 24 h immediately after incubation with the organ culture medium above-mentioned for 2 h. Both the toxins were dissolved in DMSO to obtain the stock solution, and the final concentration of DMSO in the medium was less than 0.5% (*v*/*v*). Thereupon, the supernatants were collected for LDH release measurement tests as well as the mouse IL-6, TNF-α, and IL-10 ELISA analysis as per manufacturer’s instructions, so as to choose the appropriate concentration of each toxin for further experiments. The absorbances of the samples were measured by subtracting readings at 570 nm from the readings at 450 nm using a microplate reader (Thermo Scientific, Waltham, MA, USA). Based on the results of ELISA, the optimal doses of AFM1 and OTA were selected respectively, and the same protocol above was used to detect the effect of combined AFM1 and OTA on the viability of isolated jejunal tissues as well as the stimulation of inflammatory cytokines. Finally, the intestinal tissues treated by individual AFM1 and OTA of selected concentration and the mixture of AFM1 + OTA (24 h) were collected and stored at −80 °C for RNA extraction and further RNA-seq analysis.

### 4.4. RNA-seq and Bioinformatics Analysis

Total RNA was extracted from fetal mouse jejunum tissue using Trizol reagent (Invitrogen, Carlsbad, CA, USA) according to the manufacturer’s protocols. The quality of RNA was assessed by NanoPhotometer spectrophotometer (Thermo Scientific, Waltham, MA, USA) and Agilent 2100 bioanalyzer (Agilent, Santa Clara, CA, USA). To construct the sequencing library, mRNAs were firstly enriched by Oligo (dT) beads out of total RNAs, and then fragmented into short pieces, which were subsequently transcribed to complementary DNAs (cDNAs). After purification and 3′ end-addition of adenine of cDNA, the fragments were ligated to adapters, followed by PCR amplification. The amplified and purified PCR products were finally sequenced by Illumina HiSeq2500, which was conducted in Gene Denovo Biotechnology Co., Ltd. (Guangzhou, China). Raw reads formed above were cleaned by quality control (QC), and then aligned with reference genome (*Mus musculus*, Ensembl_release 100) using HISAT2 software [85]. Afterwards, based on the above mapping results, transcripts were reconstructed and annotated by StringTie software [86]. For each transcription region, a FPKM (fragment per kilobase of transcript per million mapped reads) value was calculated to normalize and quantify its expression abundance. Further statistical analyses: sample principal component analysis (PCA), sample correlations (correlation coefficient matrix), and identification of differentially expressed genes (DEGs, via DESeq2 software) based on the thresholds: FDR < 0.05 and relative fold change > 2.0 [87], were performed individually by corresponding packages: gmodels, pheatmap, BiocManager, corrplot of R3.6.3, and visualized by ggplot2.

In this study, R package WGCNA (v1.47) was used with the aim to cluster genes into different modules according to their similarity of expression patterns (i.e., the degree of co-expression of genes) [88]. In view of this, selective condensing the huge number of candidate genes associated with toxin-induced inflammation could benefit from WGCNA study. Briefly, after removing low-quality genes, all samples were hierarchically clustered to detect any obvious outliers, and POWER value 12 was determined to ensure a scale-free network using R function pickSoftThreshold. Setting minModuleSize to 50 genes, a Dynamic Tree Cut was constructed based on correlation between expression levels of genes, and modules with high eigengene similarities (>0.75) were further merged into Merged dynamic. By calculating and comparing the degree of relationship between modules and samples, the modules strikingly affected by toxins were screened out. Thereafter, genes in the modules of interest were functionally annotated by Kyoto Encyclopedia of Genes and Genomes (KEGG) and Reactome, and then enriched into diverse significant metabolic pathways based on hypergeometric distribution algorithm. The genes involved in the biologic pathway of top significance were uploaded onto STRING database to carry out protein–protein interaction (PPI) network analysis, and the exported original data were reorganized and visualized by Cytoscape 3.6.1 to help find potential ‘hub genes’ in the network under exposure to individual and combined AFM1 and OTA.

### 4.5. FHs 74 Int Cells Culturing and Treatment

The human fetal small intestinal epithelial (FHs 74 Int) cells were cultivated in Hybri-Care Medium with 10% (*v*/*v*) FBS, antibiotics (100 U/mL penicillin, 0.1 mg/mL streptomycin), and 30 ng/mL EGF (species: human) in a humidified CO_2_ incubator (Thermo Scientific, Waltham, MA, USA) at 37 °C. FHs 74 Int cells were seeded in a 96-well plate (Corning, NY, USA) at the density of 1 × 10^4^ cells/well. After cells attached and grew up to a confluence of 90%, they were challenged with different concentrations of AFM1 (0, 1.5625, 3.125, 6.25, 12.5, 25, 50, 100 μM) or OTA (0, 0.01, 0.05, 0.1, 0.5, 1, 2, 4 μM) in serum-free medium for 48 h. The effects of AFM1 and OTA on the cellular viabilities were detected by an enhanced CCK-8 kit, and the appropriate concentration was selected for subsequent experiments. The absorbance of each well was recorded using a microplate reader (Thermo scientific, Waltham, MA, USA) at the wavelength of 450 nm. Results of treatment groups were normalized to control group as the basal line of 100%. CCK-8 assays were undertaken in three independent experiments, each with six parallel replicates per treatment.

### 4.6. RT-qPCR and ELISA Assays

FHs 74 Int cells were seeded in a 6-well plate at the density of 2 × 10^5^ cells/well, and grew up to a confluence of 90%. After that, cells were exposed to single AFM1 (12.5 and 50 μM), OTA (0.01 and 2 μM), or combination of AFM1 and OTA (12.5 μM AFM1 plus 0.01 μM OTA, or 50 μM AFM1 plus 2 μM OTA) for 48 h, respectively. Group with basal culturing medium without mycotoxins addition was set as control. According to the manufacturer’s instructions, the supernatants were collected and centrifuged to measure the concentrations of IL-6 and TNF-α by using ELISA kits. Moreover, total RNA from FHs 74 Int cells was extracted using Trizol, followed by reverse transcription to cDNA by PrimeScript RT reagent Kit (Takara, Dalian, China). The gene expressions of inflammation cytokine (*IL-6*, *TNF-α*) or inflammation-related regulator (*NFKB1*, *RelB*) were tested on the Bio-Rad CFX96 system (Bio-Rad, Hercules, CA, USA) using TB Green Premix Ex Taq Ⅱ (Takara, Dalian, China). The housekeeping gene GAPDH was used as the internal reference to calibrate inequal content of original spotting of total mRNA. The relative expression of each gene in treatment group (fold change of expression compared to control) was calculated by the method of 2^−ΔΔCt^. The primers sequences of these genes are listed in Supplementary Table S1. Furthermore, the data of qPCR of FHs 74 Int cells were compared with the corresponding data of RNA-seq of jejunal tissues of fetal mouse.

### 4.7. Western Blotting Assay

FHs 74 Int cells were cultured and treated with single AFM1, OTA or mixture of AFM1 + OTA as Section 2.6. described. Total protein, nuclear protein, and cytoplasmic protein of each sample was extracted by a ‘Western and IP cell lysis kit’ and ‘Nuclear and Cytoplasmic Protein Extraction Kit’, respectively (Beyotime, Shanghai, China), and the supernatant was collected after centrifugation. Each aliquot of protein sample was supplemented with one fifth volume of SDS loading buffer (with dithiothreitol) and then heated under 100 °C for 5 min. After that, each protein sample was loaded onto SDS–PAGE gels, transferred to membranes, and blocked for 1.5 h with blocking buffer at room temperature. Following this, the membranes were incubated overnight with primary antibodies against β-actin, PCNA, IκBα, NFKB1 and RelB, respectively (1:1000 in TBST) at 4 °C. After washing five times with TBST for 10 min, the membranes were incubated with HRP-tagged secondary antibody (1:5000 in TBST) for 1 h. The bands were detected using a Pierce™ ECL Western Blotting Substrate kit with auto-image analysis system (Tanon, Shanghai, China). The intensity of bands of IκBα, NFKB1, and RelB were further quantified by ImageJ software and normalized to that of β-actin (internal reference for total protein and cytoplasmic protein) and PCNA (internal reference for nuclear protein). The translocation of the dimer of NFKB1 and RelB from cytoplasm to nucleus was assessed by comparing their relative expression in either compartment.

### 4.8. Analysis of Intracellular ROS Concentration

FHs 74 Int cells were cultured and treated with single AFM1, OTA, or mixture of AFM1 + OTA as described in Section 2.6. The cells among groups were harvested, counted, and diluted to the same concentration with 0.01 M PBS. After that, the cells were suspended in serum-free medium containing 10 μM H2DCF-DA, incubating at 37 °C in the dark for 30 min, during which time the cells were inverted and mixed every 3–5 min. After fully removing DCFH-DA that did not enter the cells after three times washing with serum-free medium, intracellular ROS levels were detected on a microplate reader (Thermo scientific, Waltham, MA, USA) with an excitation wavelength of 488 nm and an emission wavelength of 525 nm.

### 4.9. Treatment of Antioxidant N-Acetyl-l-Cysteine (NAC) in the Presence of Toxins

FHs 74 Int cells were seeded in a 12-well plate at the density of 1 × 10^5^ cells/well and grew up to 90% confluence. The cells were divided into seven groups and incubated with control (no treated), single, and combined AFM1 (50 μM) and OTA (2 μM) with and without the presence of 5 mM NAC. After incubation at 37 °C for 48 h, ELISA kits were used to determine the levels of IL-6 and TNF-α in the supernatants following the manufacturer’s instructions.

### 4.10. Statistical Analysis

All experimental data are presented as the mean of three independent experiments ± SEM. Data analysis was performed using SPSS 19.0 (SPSS Inc., Chicago, IL, USA). Significant differences among groups were assessed using one-way ANOVA test followed by Tukey’s multiple comparisons. *p* < 0.05 was deemed to be statistically significant different between the control and treatment groups.

## Figures and Tables

**Table 1:** Top 1 pathway for each block obtained by KEGG and Reactome functional enrichment analysis.

Module	Block	Pathway
Yellow	Up	Cytokine-Cytokine receptor interaction (KEGG) GPCR ligand binding (Reactome)
	Down	Tight junction (KEGG) Tight junction interactions (Reactome)
Blue up	1	Cytokine-Cytokine receptor interaction (KEGG) Cytokine signaling in immune system (Reactome)
	2	Neuroactive ligand-receptor interaction (KEGG) GPCR signaling (Reactome)
	3	Ribosome (KEGG) Translation (Reactome)
	4	Oxidative phosphorylation (KEGG) Respiratory electron transport (Reactome)
	5	Calcium signaling pathway (KEGG) Muscle contraction (Reactome)
	6	Systemic lupus erythematosus (KEGG) DNA methylation (Reactome)
	7	ECM-receptor interaction (KEGG) ECM proteoglycans (Reactome)
	8	Folate biosynthesis (KEGG) Post-translational modification (Reactome)
	9	Phagosome (KEGG) Golgi-to-ER retrograde transport (Reactome)

## Data Availability

Data are available upon request, please contact the contributing authors.

## Supplementary Materials

The following are available online at https://www.mdpi.com/article/10.3390/toxins14030173/s1, Figure S1: Effects of (A) various doses of AFM1, (B) various doses OTA and (C) AFM1 + OTA treatment for 24 h on the release of IL-10 from the isolated jejunal tissues. In panel (C), the concentration of AFM1 and OTA was 50 μM and 4 μM, respectively. Results were shown as mean ± SEM (n ≥ 6). * *p* < 0.05 statistically significantly compared with control, Figure S2: FPKM distribution of all samples, Figure S3: Relative expression of each gene (vs. DMSO group) involved in block 1 ‘Cytokine-Cytokine receptor interaction’ pathway, Figure S4: Relative gene expression (vs. DMSO group) of (A) *IL6* (B) *TNF-α* (C) *NFKB1* and (D) *RelB* using qPCR assay in FHs 74 Int cells and RNA-seq method in jejunal tissues of fetal mice. Blue bar and red bar represent the qPCR-data from toxin groups at low and high concentration, respectively (AFM1 low: 12.5 μM, AFM1 high: 50 μM; OTA low: 0.01 μM; OTA high: 2 μM). Green fold line represents the data from RNA-seq, Table S1: List of primer sequence for Real-Time qPCR, Table S2: List of several genes adjacent to NFKB1 based on PPI network analysis. Different lowercase indicates statistical differences between groups (*p* < 0.05).

## References

[1] Pitt J.I., Basilico J.C., Abarca M.L., Lopez C. (2000). Mycotoxins and toxigenic fungi. Med. Mycol..

[2] Hernandez M., Juan-Garcia A., Molto J.C., Manes J., Juan C. (2021). Evaluation of Mycotoxins in Infant Breast Milk and Infant Food, Reviewing the Literature Data. Toxins.

[3] Juan C., Raiola A., Mañes J., Ritieni A. (2014). Presence of mycotoxin in commercial infant formulas and baby foods from Italian market. Food Control.

[4] Raiola A., Tenore G.C., Manyes L., Meca G., Ritieni A. (2015). Risk analysis of main mycotoxins occurring in food for children: An overview. Food Chem. Toxicol..

[5] Ezekiel C., Abia W., Braun D., Sarkanj B., Ayeni K., Oyedele O., Michael-Chikezie E., Ezekiel V., Mark B., Ahuchaogu C. (2020). Comprehensive mycotoxin exposure biomonitoring in breastfed and non-exclusively breastfed Nigerian children. medRxiv.

[6] Amuzie C., Bandyopadhyay R., Bhat R., Black R., Burger H.-M., Cardwell K., Gelderblom W., Gong Y.Y., Groopman J., Kimanya M. (2015). Mycotoxin Control in Low- and Middle-Income Countries.

[7] Gong Y.Y., Watson S., Routledge M. (2016). Aflatoxin Exposure and Associated Human Health Effects, a Review of Epidemiological Studies. Food Saf..

[8] Alassane-Kpembi I., Schatzmayr G., Taranu I., Marin D., Puel O., Oswald I.P. (2017). Mycotoxins co-contamination: Methodological aspects and biological relevance of combined toxicity studies. Crit. Rev. Food Sci. Nutr..

[9] Assunção R., Silva M., Alvito P. (2016). Challenges in risk assessment of multiple mycotoxins in food. World Mycotoxin J..

[10] Chen C., Mitchell N.J., Gratz J., Houpt E.R., Gong Y., Egner P.A., Groopman J.D., Riley R.T., Showker J.L., Svensen E. (2018). Exposure to aflatoxin and fumonisin in children at risk for growth impairment in rural Tanzania. Environ. Int..

[11] De Santis B., Brera C., Mezzelani A., Soricelli S., Ciceri F., Moretti G., Debegnach F., Bonaglia M.C., Villa L., Molteni M. (2019). Role of mycotoxins in the pathobiology of autism: A first evidence. Nutr. Neurosci..

[12] De Santis B., Raggi M.E., Moretti G., Facchiano F., Mezzelani A., Villa L., Bonfanti A., Campioni A., Rossi S., Camposeo S. (2017). Study on the Association among Mycotoxins and other Variables in Children with Autism. Toxins.

[13] Katerere D., Shephard G., Faber M. (2008). Infant malnutrition and chronic aflatoxicosis in Southern Africa: Is there a link?. Int. J. Food Saf. Nutr. Public Health.

[14] Smith L.E., Prendergast A.J., Turner P.C., Mbuya M.N.N., Mutasa K., Kembo G., Stoltzfus R.J., for the Sanitation Hygiene Infant Nutrition Efficacy (SHINE) Trial Team (2015). The Potential Role of Mycotoxins as a Contributor to Stunting in the SHINE Trial. Clin. Infect. Dis..

[15] Wood L., Wood M., Fisher B., Jaspan H., Sodora D. (2017). T Cell Activation in South African HIV-Exposed Infants Correlates with Ochratoxin A Exposure. Front. Immunol..

[16] Alegbe S., Yakubu S., Olonitola O.S., Mukhtar M. (2018). Assessing Aflatoxin M1 levels among lactating mothers’ in Damaturu Yobe state, Nigeria. Bayero J. Pure Appl. Sci..

[17] Eshete M., Gebremedhin S., Alemayehu F.R., Taye M., Boshe B., Stoecker B.J. (2021). Aflatoxin contamination of human breast milk and complementary foods in southern Ethiopia. Matern. Child Nutr..

[18] Hooshfar S., Khosrokhavar R., Yazdanpanah H., Eslamizad S., Kobarfard F., Nazari F., Kokaraki V., Kokkinakis M., Goumenou M., Tsitsimpikou C. (2020). Health risk assessment of aflatoxin M1 in infant formula milk in IR Iran. Food Chem. Toxicol..

[19] Islam F., Das Trisha A., Hafsa J.M., Hasan A., Degen G.H., Ali N. (2021). Occurrence of aflatoxin M1 in human breast milk in Bangladesh. Mycotoxin Res..

[20] Kang’ethe E., Gatwiri M., Sirma A., Ouko O., Mburugu-Musoti C., Kitala P., Gitahi N., Nderitu J., Mung’atu J., Hietaniemi V. (2017). Exposure of Kenyan population to aflatoxins in foods with special reference to Nandi and Makueni counties. Food Qual. Saf..

[21] Cherkani-Hassani A., Ghanname I., Zinedine A., Sefrioui H., Qmichou Z., Mouane N. (2020). Aflatoxin M1 prevalence in breast milk in Morocco: Associated factors and health risk assessment of newborns “CONTAMILK study”. Toxicon.

[22] Karayagiz Muslu G., Ozdemir M. (2020). Occurrence of and Factors Associated With the Presence of Aflatoxin M1 in Breast Milk of Mothers in Fethiye, Turkey. Biol. Res. Nurs..

[23] Quevedo-Garza P.A., Amador-Espejo G.G., Salas-Garcia R., Ramos-Pena E.G., Trujillo A.J. (2020). Aflatoxin M1 Determination in Infant Formulae Distributed in Monterrey, Mexico. Toxins.

[24] Gong Y.Y., Turner P., Hall A.J., Wild C.P. (2008). Aflatoxin exposure and impaired child growth in West Africa: An unexplored international public health burden. Mycotoxins Detect. Methods Manag. Public Health Agric. Trade.

[25] Magoha H., Kimanya M., De Meulenaer B., Roberfroid D., Lachat C., Kolsteren P. (2016). Risk of dietary exposure to aflatoxins and fumonisins in infants less than 6 months of age in Rombo, Northern Tanzania. Matern. Child Nutr..

[26] Okoth S.A., Ohingo M. (2004). Dietary aflatoxin exposure and impaired growth in young children from Kisumu District, Kenya: Cross sectional study. Afr. J. Health Sci..

[27] Smith L., Stoltzfus R., Prendergast A. (2012). Food Chain Mycotoxin Exposure, Gut Health, and Impaired Growth: A Conceptual Framework. Adv. Nutr..

[28] Ali N., Muñoz K., Degen G. (2017). Ochratoxin A and its metabolites in urines of German adults—An assessment of variables in biomarker analysis. Toxicol. Lett..

[29] Bondy G., Curran I., Coady L., Armstrong C., Bourque C., Bugiel S., Caldwell D., Kwong K., Lefebvre D., Maurice C. (2021). A one-generation reproductive toxicity study of the mycotoxin ochratoxin A in Fischer rats. Food Chem. Toxicol..

[30] Hassan A., Sheashaa H., Fattah M., Ibrahim A., Gaber O., Sobh M. (2006). Study of Ochratoxin A as an Environmental Risk That Causes Renal Injury in Breast-Fed Egyptian Infants. Pediatr. Nephrol..

[31] Khoshnamvand Z., Nazari F., Mehrasbi M., Hosseini M.-J. (2019). Occurrence and Safety Evaluation of Ochratoxin A in Cereal-based Baby Foods Collected from Iranian Retail Market. J. Food Sci..

[32] Cappozzo J., Jackson L., Lee H.J., Zhou W., Al-Taher F., Zweigenbaum J., Ryu D. (2017). Occurrence of Ochratoxin A in Infant Foods in the United States. J. Food Prot..

[33] Sun X., Fu X., Zhu M. (2018). Ex vivo gut culture for studying differentiation and migration of small intestinal epithelial cells. Open Biol..

[34] Gleeson J., Fein K., Chaudhary N., Doerfler R., Newby A., Whitehead K. (2021). The enhanced intestinal permeability of infant mice enables oral protein and macromolecular absorption without delivery technology. Int. J. Pharm..

[35] Gasparoni A., Ciardelli L., Avanzini A., Castellazzi A., Carini R., Rondini G., Chirico G. (2003). Age-Related Changes in Intracellular Th1/Th2 Cytokine Production, Immunoproliferative T Lymphocyte Response and Natural Killer Cell Activity in Newborns, Children and Adults. Biol. Neonate.

[36] Leushacke M., Barker N. (2014). Ex vivo culture of the intestinal epithelium: Strategies and applications. Gut.

[37] Zheng N., Gao Y., Zhu W., Meng D., Walker W. (2020). Short chain fatty acids produced by colonizing intestinal commensal bacterial interaction with expressed breast milk are anti-inflammatory in human immature enterocytes. PLoS ONE.

[38] Blackwood B., Wood D., Yuan C., Nicolas J., Plaen I., Farrow K., Md P., Turner J., Hunter C. (2016). A Role for cAMP and Protein Kinase A in Experimental Necrotizing Enterocolitis. Am. J. Pathol..

[39] Liu T., Zhang L., Joo D., Sun S.-C. (2017). NF-κB signaling in inflammation. Signal Transduct. Target. Ther..

[40] Martins G.R., Gelaleti G.B., Moschetta M.G., Maschio-Signorini L.B., Zuccari D.A.P.d.C. (2016). Proinflammatory and Anti-Inflammatory Cytokines Mediated by NF-κB Factor as Prognostic Markers in Mammary Tumors. Mediat. Inflamm..

[41] Wopereis H., Oozeer R., Knipping K., Belzer C., Knol J. (2014). The first thousand days—Intestinal microbiology of early life: Establishing a symbiosis. Pediatr. Allergy Immunol..

[42] Ossa J.C., Yanez D., Valenzuela R., Gallardo P., Lucero Y., Farfan M.J. (2018). Intestinal Inflammation in Chilean Infants Fed With Bovine Formula vs. Breast Milk and Its Association With Their Gut Microbiota. Front. Cell Infect. Microbiol..

[43] Arrieta M.C., Arevalo A., Stiemsma L., Dimitriu P., Chico M.E., Loor S., Vaca M., Boutin R.C.T., Morien E., Jin M. (2018). Associations between infant fungal and bacterial dysbiosis and childhood atopic wheeze in a nonindustrialized setting. J. Allergy Clin. Immunol..

[44] Maffeis C., Martina A., Corradi M., Quarella S., Nori N., Torriani S., Plebani M., Contreas G., Felis G.E. (2016). Association between intestinal permeability and faecal microbiota composition in Italian children with beta cell autoimmunity at risk for type 1 diabetes. Diabetes Metab. Res. Rev..

[45] Orivuori L., Mustonen K., de Goffau M.C., Hakala S., Paasela M., Roduit C., Dalphin J.C., Genuneit J., Lauener R., Riedler J. (2015). High level of fecal calprotectin at age 2 months as a marker of intestinal inflammation predicts atopic dermatitis and asthma by age 6. Clin. Exp. Allergy.

[46] Kang R., Li R., Dai P., Li Z., Li Y., Li C. (2019). Deoxynivalenol induced apoptosis and inflammation of IPEC-J2 cells by promoting ROS production. Environ. Pollut..

[47] Liao P., Li Y., Li M., Chen X., Yuan D., Tang M., Xu K. (2020). Baicalin alleviates deoxynivalenol-induced intestinal inflammation and oxidative stress damage by inhibiting NF-kappaB and increasing mTOR signaling pathways in piglets. Food Chem. Toxicol..

[48] Tang M., Yuan D., Liao P. (2021). Berberine improves intestinal barrier function and reduces inflammation, immunosuppression, and oxidative stress by regulating the NF-kappaB/MAPK signaling pathway in deoxynivalenol-challenged piglets. Environ. Pollut..

[49] Ying C., Hong W., Nianhui Z., Chunlei W., Kehe H., Cuiling P. (2019). Nontoxic concentrations of OTA aggravate DON-induced intestinal barrier dysfunction in IPEC-J2 cells via activation of NF-kappaB signaling pathway. Toxicol. Lett..

[50] Yu Y.H., Lai Y.H., Hsiao F.S., Cheng Y.H. (2021). Effects of Deoxynivalenol and Mycotoxin Adsorbent Agents on Mitogen-Activated Protein Kinase Signaling Pathways and Inflammation-Associated Gene Expression in Porcine Intestinal Epithelial Cells. Toxins.

[51] Ruan D., Wang W.C., Lin C.X., Fouad A.M., Chen W., Xia W.G., Wang S., Luo X., Zhang W.H., Yan S.J. (2019). Effects of curcumin on performance, antioxidation, intestinal barrier and mitochondrial function in ducks fed corn contaminated with ochratoxin A. Animal.

[52] Tong C., Li P., Yu L.-H., Li L., Li K., Chen Y., Yang S.-H., Long M. (2020). Selenium-rich yeast attenuates ochratoxin A-induced small intestinal injury in broiler chickens by activating the Nrf2 pathway and inhibiting NF-KB activation. J. Funct. Foods.

[53] Gao Y., Ye Q., Bao X., Huang X., Wang J., Zheng N. (2020). Transcriptomic and proteomic profiling reveals the intestinal immunotoxicity induced by aflatoxin M1 and ochratoxin A. Toxicon.

[54] Xu X., Yan G., Chang J., Wang P., Yin Q., Liu C., Zhu Q., Lu F. (2020). Comparative Transcriptome Analysis Reveals the Protective Mechanism of Glycyrrhinic Acid for Deoxynivalenol-Induced Inflammation and Apoptosis in IPEC-J2 Cells. Oxidative Med. Cell. Longev..

[55] Leong H.S., Kipling D. (2009). Text-based over-representation analysis of microarray gene lists with annotation bias. Nucleic Acids Res..

[56] Zhang H., Deng X., Zhou C., Wu W., Zhang H. (2020). Deoxynivalenol Induces Inflammation in IPEC-J2 Cells by Activating P38 Mapk And Erk1/2. Toxins.

[57] Yang X., Gao Y., Yan Q., Bao X., Zhao S., Wang J., Zheng N. (2019). Transcriptome Analysis of Ochratoxin A-Induced Apoptosis in Differentiated Caco-2 Cells. Toxins.

[58] Concetti J., Wilson C.L. (2018). NFKB1 and Cancer: Friend or Foe?. Cells.

[59] Gasparini C., Foxwell B.M., Feldmann M. (2009). RelB/p50 regulates CCL19 production, but fails to promote human DC maturation. Eur. J. Immunol..

[60] Moriwaki K., Balaji S., McQuade T., Malhotra N., Kang J., Chan F.K.-M. (2014). The necroptosis adaptor RIPK3 promotes injury-induced cytokine expression and tissue repair. Immunity.

[61] Huang L., Zhao Z., Duan C., Wang C., Zhao Y., Yang G., Gao L., Niu C., Xu J., Li S. (2019). Lactobacillus plantarum C88 protects against aflatoxin B(1)-induced liver injury in mice via inhibition of NF-κB-mediated inflammatory responses and excessive apoptosis. BMC Microbiol..

[62] Wang X., Zhang Y., Zhao J., Cao L., Zhu L., Huang Y., Chen X., Rahman S.U., Feng S., Li Y. (2019). Deoxynivalenol Induces Inflammatory Injury in IPEC-J2 Cells via NF-κB Signaling Pathway. Toxins.

[63] Ringot D., Chango A., Schneider Y.-J., Larondelle Y. (2006). Toxicokinetics and toxicodynamics of ochratoxin A, an update. Chem. Biol. Interact..

[64] Tran V.N., Viktorová J., Ruml T. (2020). Mycotoxins: Biotransformation and Bioavailability Assessment Using Caco-2 Cell Monolayer. Toxins.

[65] Tao Y., Xie S., Xu F., Liu A., Wang Y., Chen D., Pan Y., Huang L., Peng D., Wang X. (2018). Ochratoxin A: Toxicity, oxidative stress and metabolism. Food Chem. Toxicol..

[66] Bbosa G., Kitya D., Lubega A., Ogwal-Okeng J., Anokbonggo W., Kyegombe D. (2013). Review of the Biological and Health Effects of Aflatoxins on Body Organs and Body Systems. Aflatoxins Recent Adv. Future Prospect..

[67] Li H., Li S., Yang H., Wang Y., Wang J., Zheng N. (2019). l-Proline Alleviates Kidney Injury Caused by AFB1 and AFM1 through Regulating Excessive Apoptosis of Kidney Cells. Toxins.

[68] Boesch-Saadatmandi C., Loboda A., Jozkowicz A., Huebbe P., Blank R., Wolffram S., Dulak J., Rimbach G. (2008). Effect of ochratoxin A on redox-regulated transcription factors, antioxidant enzymes and glutathione-S-transferase in cultured kidney tubulus cells. Food Chem. Toxicol..

[69] Cavin C., Delatour T., Marin-Kuan M., Holzhäuser D., Higgins L., Bezençon C., Guignard G., Junod S., Richoz-Payot J., Gremaud E. (2007). Reduction in Antioxidant Defenses may Contribute to Ochratoxin A Toxicity and Carcinogenicity. Tox-Icological Sci..

[70] Lan M., Zhang Y., Wan X., Pan M.-H., Xu Y., Sun S.-C. (2020). Melatonin ameliorates ochratoxin A-induced oxidative stress and apoptosis in porcine oocytes. Environ. Pollut..

[71] Forrester S.J., Kikuchi D.S., Hernandes M.S., Xu Q., Griendling K.K. (2018). Reactive Oxygen Species in Metabolic and Inflammatory Signaling. Circ. Res..

[72] Shi X.-Z., Lindholm P.F., Sarna S.K. (2003). NF-κB activation by oxidative stress and inflammation suppresses contractility in colonic circular smooth muscle cells. Gastroenterology.

[73] Su L.-J., Zhang J.-H., Gomez H., Murugan R., Hong X., Xu D., Jiang F., Peng Z.-Y. (2019). Reactive Oxygen Species-Induced Lipid Peroxidation in Apoptosis, Autophagy, and Ferroptosis. Oxidative Med. Cell. Longev..

[74] Yang S., Lian G. (2020). ROS and diseases: Role in metabolism and energy supply. Mol. Cell. Biochem..

[75] Kelley N., Jeltema D., Duan Y., He Y. (2019). The NLRP3 Inflammasome: An Overview of Mechanisms of Activation and Regulation. Int. J. Mol. Sci..

[76] Vajjhala P.R., Mirams R.E., Hill J.M. (2012). Multiple binding sites on the pyrin domain of ASC protein allow self-association and interaction with NLRP3 protein. J. Biol. Chem..

[77] Swanson K.V., Deng M., Ting J.P.Y. (2019). The NLRP3 inflammasome: Molecular activation and regulation to therapeutics. Nat. Rev. Immunol..

[78] Khatri V., Kalyanasundaram R. (2021). Therapeutic implications of inflammasome in inflammatory bowel disease. FASEB J..

[79] Zhen Y., Zhang H. (2019). NLRP3 Inflammasome and Inflammatory Bowel Disease. Front. Immunol..

[80] Kowalska K., Habrowska-Górczyńska D.E., Domińska K., Urbanek K.A., Piastowska-Ciesielska A.W. (2020). ERβ and NFκB—Modulators of Zearalenone-Induced Oxidative Stress in Human Prostate Cancer Cells. Toxins.

[81] Liu Q., Wen J., Zhu J., Zhang T., Deng Y., Jiang J. (2019). Aromatic hydrocarbon receptor regulates chicken cytochrome P450 1A5 transcription: A novel insight into T-2 toxin-induced gene expression and cytotoxicity in LMH cells. Biochem. Pharmacol..

[82] Grenier B., Oswald I. (2011). Mycotoxin co-contamination of food and feed: Meta-Analysis of publications describing toxicological interactions. World Mycotoxin J..

[83] Tavares A., Alvito P., Loureiro S., Louro H., Silva M. (2013). Multi-mycotoxin determination and in vitro combined cytotoxic effects of aflatoxin M1 and ochratoxin A. World Mycotoxin J..

[84] Meng D., Zhu W., Shi H.N., Lu L., Wijendran V., Xu W., Walker W.A. (2015). Toll-like receptor-4 in human and mouse colonic epithelium is developmentally regulated: A possible role in necrotizing enterocolitis. Pediatr. Res..

[85] Kim D., Langmead B., Salzberg S.L. (2015). HISAT: A fast spliced aligner with low memory requirements. Nat. Methods.

[86] Pertea M., Pertea G.M., Antonescu C.M., Chang T.C., Mendell J.T., Salzberg S.L. (2015). StringTie enables improved reconstruction of a transcriptome from RNA-seq reads. Nat. Biotechnol..

[87] Love M.I., Huber W., Anders S. (2014). Moderated estimation of fold change and dispersion for RNA-seq data with DESeq2. Genome Biol..

[88] Langfelder P., Horvath S. (2008). WGCNA: An R package for weighted correlation network analysis. BMC Bioinform..

