# Supplementary material for: Ex Vivo and In Vitro Studies Revealed Underlying Mechanisms of Immature Intestinal Inflammatory Responses Caused by Aflatoxin M1 Together with Ochratoxin A
Source: Toxins (Basel). 2022 Feb 25;14(3):173. doi: 10.3390/toxins14030173 (PMC8953104; doi:10.3390/toxins14030173)
Supplement: Supplementary file 1 [file toxins-14-00173-s001.zip › toxins-1565453-supplementary.pdf]

# Supplementary Materials: Ex Vivo and In Vitro Studies Revealed Underlying Mechanisms of Immature Intestinal Inflammatory Responses Caused by Aflatoxin M1 Together with Ochratoxin A

**Zi-Wei Wang, Ya-Nan Gao, Sheng-Nan Huang, Jia-Qi Wang and Nan Zheng**

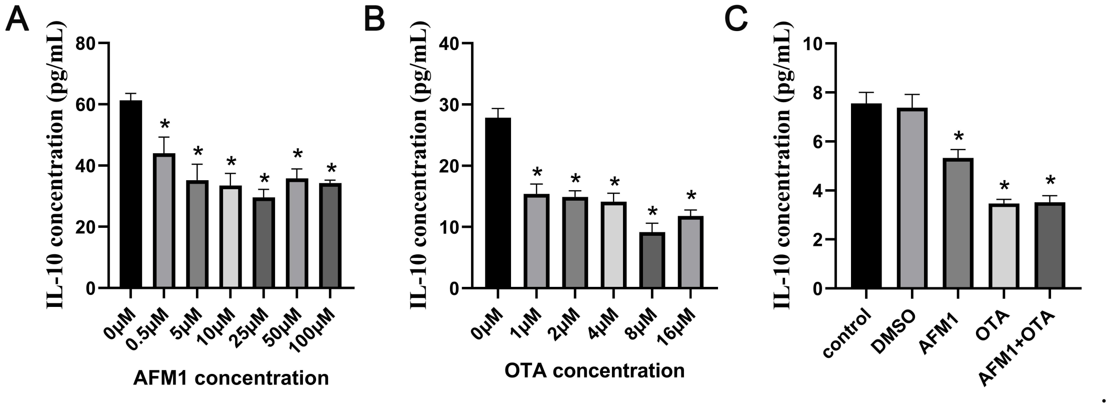


**Figure S1.** Effects of (A) various doses of AFM1, (B) various doses OTA and (C) AFM1+OTA treatment for 24h on the release of IL-10 from the isolated jejunal tissues. In panel C, the concentration of AFM1 and OTA was 50 μM and 4 μM, respectively. Results were shown as mean ± SEM (n ≥ 6). * $p < 0.05$ statistically significantly compared with control.

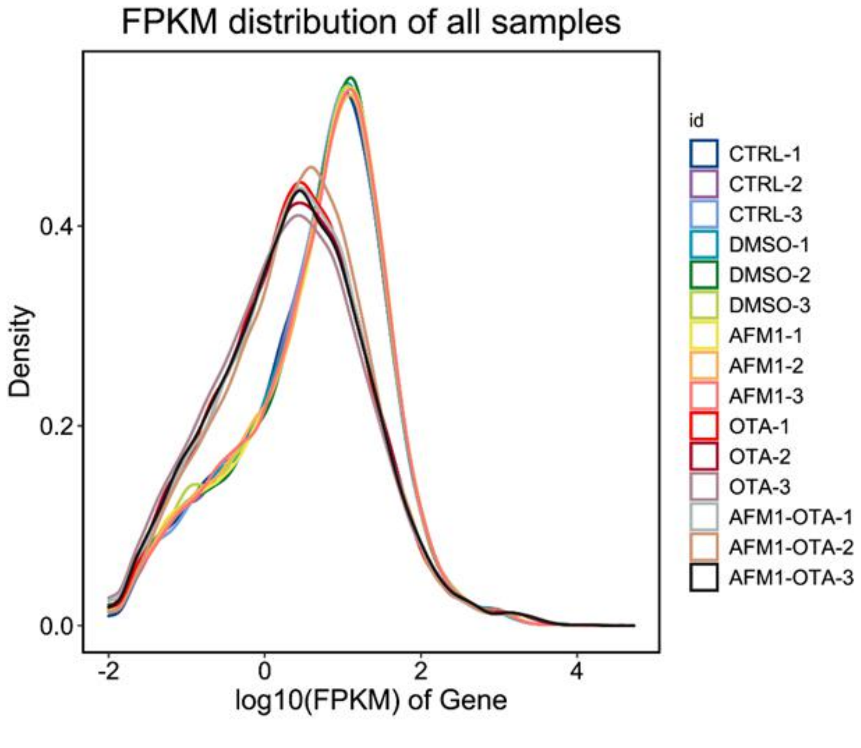


**Figure S2.** FPKM distribution of all samples.

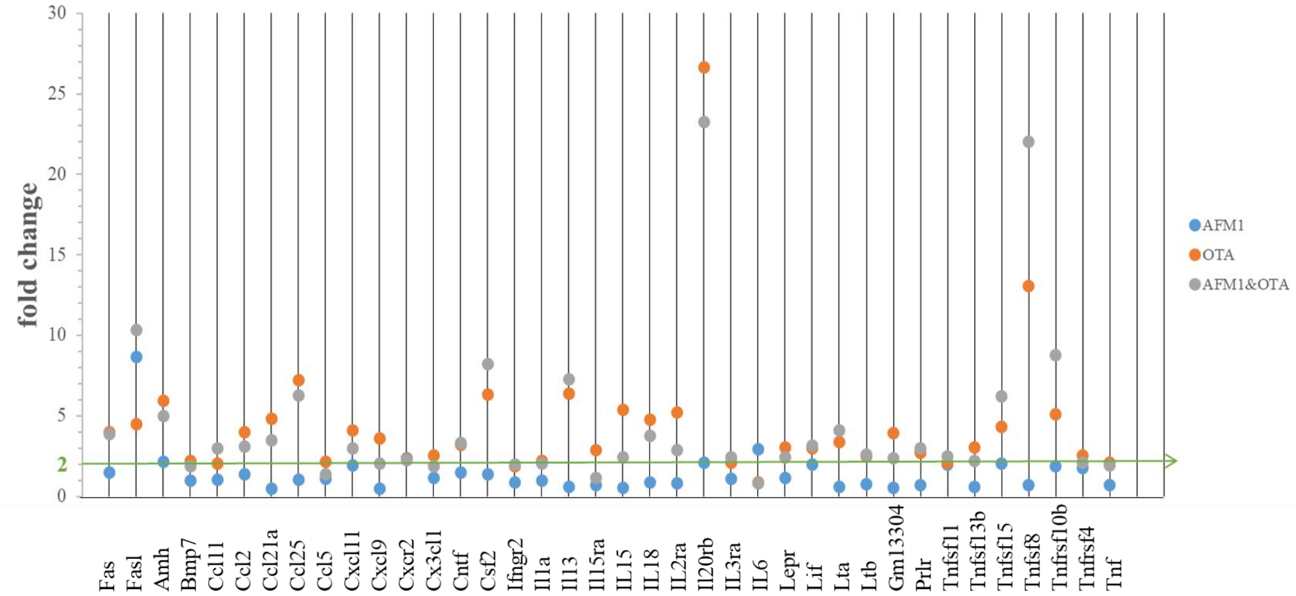


**Figure S3.** Relative expression of each gene (vs. DMSO group) involved in block 1 'Cytokine-Cytokine receptor interaction' pathway.

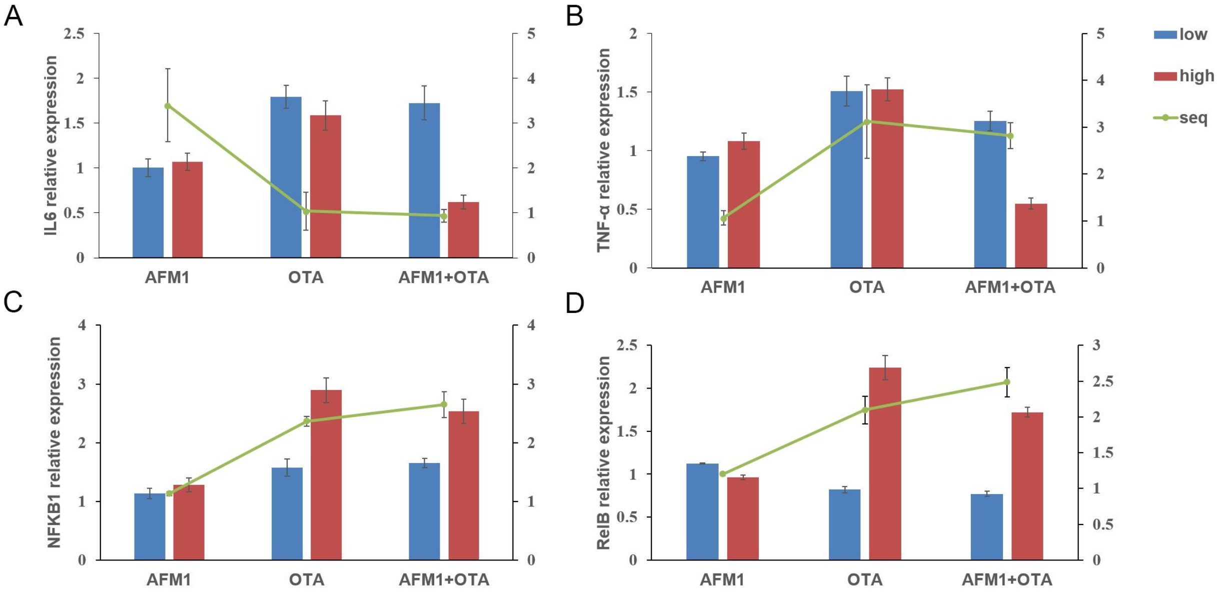


**Figure S4.** Relative gene expression (vs. DMSO group) of (A) *IL6* (B) *TNF-α* (C) *NFKB1* and (D) *RelB* using qPCR assay in FHs 74 Int cells and RNA-seq method in jejunal tissues of fetal mice. Blue bar and red bar represent the qPCR-data from toxin groups at low and high concentration, respectively (AFM1 low: 12.5 μM, AFM1 high: 50 μM; OTA low: 0.01 μM; OTA high: 2 μM). Green fold line represents the data from RNA-seq.

**Table S1.** List of primer sequence for Real-Time qPCR.

| Gene | Sequence (5' to 3') | |
|---|---|---|
| GAPDH (human) | F | GCAAGAGCACAAGAGGAAGAG |
| | R | TCTACATGGCAACTGTGAGGA |
| IL-6 (human) | F | CCTTCCAAAGATGGCTGAAA |
| | R | CAGGGGTGGTTATTGCATCT |
| TNF-$\alpha$ (human) | F | CTGAACTTCGGGGTGATCG |
| | R | GCTTGGTGGTTTGCTACGAC |
| NFKB1 (human) | F | AACAGAGAGGATTTCGTTTCCG |
| | R | TTTGACCTGAGGGTAAGACTTCT |
| RelB (human) | F | CATTGAGCGGAAGATTCAAC |
| | R | GCAGCTCTGATGTGTTTGTG |

**Table S2.** List of several genes adjacent to NFKB1.

| Gene name | Protein description | Fold change vs. DMSO group | | | |
|---|---|---|---|---|---|
| | | **DMSO** | **AFM1** | **OTA** | **AFM1+OTA** |
| Nlrp3 | NLR family, pyrin domain containing 3; the sensor component of the inflammasome, plays a crucial role in innate immunity and inflammation | 1.00 [b] | 3.16 [b] | 4.98 [ab] | 7.54 [a] |
| Pycard | PYD and CARD domain containing; Functions as key mediator in apoptosis and inflammation; Required for recruitment of caspase-1 to inflammasomes containing certain pattern recognition receptors, e.g., Nlrp3 | 1.00 [b] | 0.83 [b] | 3.28 [a] | 3.86 [a] |
| Casp1 | Besides apoptotic regulation, also plays a key role in cell immunity as an inflammatory response initiator: once activated through formation of an inflammasome complex, it initiates a proinflammatory response through the cleavage of the two inflammatory cytokines pro-IL1B and pro-IL18, releasing the mature cytokines | 1.00 | 0.73 | 2.23 | 2.16 |
| Il1b | interleukin 1 beta | 1.00 [b] | 8.27 [a] | 0.68 [b] | 0.18 [b] |
| Il1a | interleukin 1 alpha | 1.00 | 0.98 | 2.22 | 2.07 |
| Il18 | interleukin 18 | 1.00 [b] | 0.88 [b] | 4.77 [a] | 3.79 [a] |
| Tnf | tumor necrosis factor | 1.00 | 0.73 | 2.13 | 1.93 |

Note: Different lowercase indicates statistical differences between groups ($p < 0.05$).
